# Mucoepidermoid carcinoma arising in Warthin’s tumor of the parotid gland: Clinicopathological characteristics and immunophenotypes

**DOI:** 10.1038/srep30149

**Published:** 2016-07-15

**Authors:** Chunkai Yu, Zhigang Song, Zhibo Xiao, Qiushi Lin, Xiaoqun Dong

**Affiliations:** 1 Department of Pathology, Beijing Shijitan Hospital, Capital Medical University, Beijing 100038, P. R. China; 2 Department of Pathology, The Chinese PLA General Hospital, Beijing 100853, P. R. China; 3 Department of Plastic Surgery, The Second Affiliated Hospital of Harbin Medical University, P. R. China; 4 Department of Gastroenterology, Stephenson Cancer Center, Department of Internal Medicine, College of Medicine, The University of Oklahoma Health Sciences Center, USA

## Abstract

Mucoepidermoid carcinoma (MEC), an extremely rare tumor, arises from the epithelial component of preexisting parotid Warthin tumors (WT). Among the 309 cases of surgically resected WTs in Chinese PLA General Hospital and Beijing Shijitan Hospital of Capital Medical University, 5 cases (1.6%) fulfilled the criteria for MECs transformed from WTs. Clinicopathological characteristics of MECs was demonstrated in order to avoid misdiagnosis of this rare type of tumor. All the 5 patients, 3 males and 2 females, presented painless masses in the parotid gland. MECs were located inside or at the edge of WTs, with an obvious transitional zone between WT and MEC. Basal cells of WTs and epidermoid cells of MECs were strongly positive for cytokeratin CK5/6, CK34βE12, and P63; whereas negative for CK7, CK20, and CEA. Mucous cells of MECs were positive for CK7, CEA, as well as periodic acid-Schiff (PAS), whereas negative for CK5/6, CK34βE12, CK20, and P63. MECs patients were followed up for 25–69 months after surgery, presenting no evidence of recurrence or metastasis. Collectively, MECs arising from WT is very rare. The pathological diagnosis was based on histological morphology, especially the transitional zone between WT and MEC.

Warthin’s tumor (WT), also known as papillary cystadenoma lymphomatosum, monomorphic adenoma or adenolymphoma, cystadenolymphoma or epitheliolymphoid cyst, which consists of lymphoid stroma and glandular epithelium with characteristic eosinophilic cytoplasm, is a benign tumor of the salivary glands. It is the second most common benign tumor of the parotid gland after pleomorphic adenoma, representing 5–11% of primary tumors in salivary glands^1^,^2^. Warthin’s tumor arises mainly in the parotid gland synchronously or metachronously in the same or contralateral gland. It affects mainly males with age of onset at 60–70 years. An increasing incidence for females has been reported in China. The etiology of Warthin’s tumor is unclear; progesterone receptors and smoking may be associated with individual susceptibility to this tumor.

Usually, Warthin tumor presents as a slowly growing, freely moveable, painless soft tissue mass located in the superficial lobe of the parotid gland^3^, which can be revealed by Ultrasound as a well-defined hypoechoic mass^4^. Diagnosis is often confirmed by a fine-needle aspiration biopsy (FNAB).

Surgical resection is the major treatment of Warthin tumor, however, there is contradictory opinions on the appropriate extent of surgery^3^. It was proposed that limited excisions including enucleation and removal of the inferior half of the superficial lobe are sufficient^5^,^6^. Other investigators argued that superficial parotidectomy is standard^7^. Manifestation of a tumor in the inner lobe requires total parotidectomy.

Malignant transformation of Warthin’s tumor is more common in the lymphoid component than the epithelial component, although malignant carcinoma is very rare (0.3%)^8^. Carcinomatous components have been reported as mucoepidermoid carcinoma (MEC)^9^,^10^,^11^,^12^,^13^, squamous cell carcinoma (the most common)^8^,^14^, oncocytic carcinoma, adenocarcinoma^15^,^16^,^17^,^18^ and undifferentiated carcinoma^19^,^20^.

Only 19 cases of WT co-existing with MEC have been reported worldwide^7^. Here we reported 5 new cases of MECs arising in Warthin’s tumor of the parotid gland. The purpose of this study was to improve the understanding of clinical and pathological characteristics of MECs, and thus to avoid misdiagnosis of this very rare type of tumor.

## Results

### Clinicopathological characteristics

**Figure 1:**
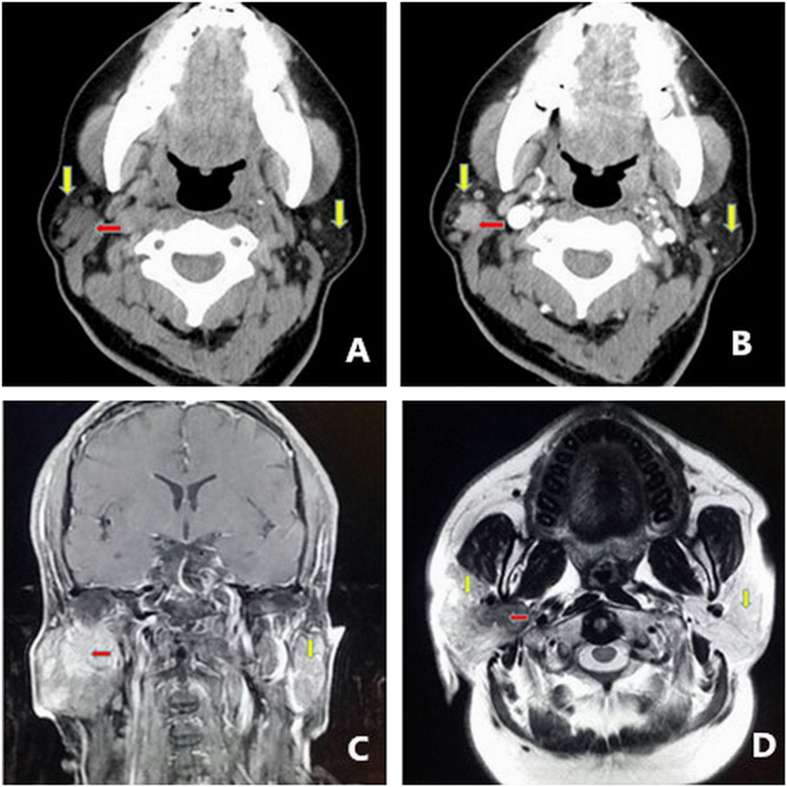
Major imaging findings for patients. (**A**) CT scanning in case 1 displayed circular nodule shadow in the right parotid gland. (**B**) Enhanced scanning identified cystic or necrotic tumor. (**C**) MRI displayed irregular shaped lesions in the right parotid gland globe in Case #5. (**D**) MRI enhanced scan revealed MECs tumors (yellow arrow highlighted parotid gland, red arrow highlighted the tumor).

The demographic, clinical and pathological information of MECs patients were summarized in Table 1. Among these 5 cases, 3 males and 2 females; the median age was 43 years (range 26–63 years). All patients presented painless masses in the parotid gland region, except for Case 5, whose first symptom was progressive facial paralysis. Four cases developed tumors in the superficial lobe of the parotid gland, and 1 case in the deep lobe. In Case #1, CT scanning displayed circular nodule shadow with slightly high density in the right parotid gland, with clear boundary and multiple nodules surrounding the shadow (Fig. 1A). Enhanced CT scanning described lesions in moderate strengthening, with lower density inside an oval shaped area, which was considered as internal cystic or necrosis of the tumor (Fig. 1B). In Case #5, MRI imaging displayed multiple long T-1 and short T-2 signals varying in sizes in the right parotid gland globe with irregular shapes (Fig. 1C). MRI enhanced scanning could reveal moderate enhancement of MECs tumors (Fig. 1D). Three cases received single surgery treatment, and 2 cases received surgery combined with radioactive particle implantation.

### Gross appearance

**Figure 2:**
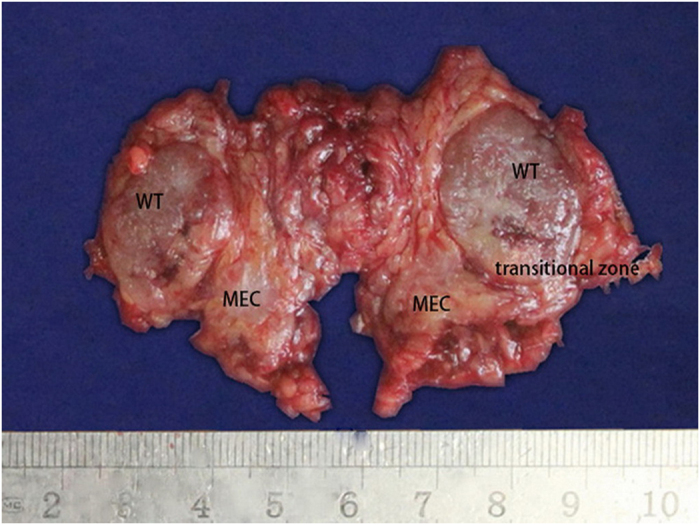
Gross appearance of the tumor derived from Case #5. Solid sections were highlighted in grayish-yellow or grayish-brown color. Tumor had invaded the outside border of the capsule.

Each mass exhibited a nodular or oval shape. There were multiple tumors (ranged from 0.5–2.5 cm in diameter) in Case #5. The tumor size of another 4 cases ranged from 2 cm to 4.2 cm in the maximum diameter (Table 1). The masses of Cases #2 and #3 were completely encapsulated with clear boundaries. Masses of the other cases were mostly encapsulated with ambiguous boundary to the surrounding parotid gland tissues. Cross sections of the masses in Cases #1, 3, and 4 were solid, presenting grayish-yellow or grayish-brown color. Cross section of the mass in Case #2 was cystic-solid, with mucous fluid in the cystic areas. In Case #5, the mass had invaded the outside border of the capsule (Fig. 2).

### Microscopic features

**Figure 3:**
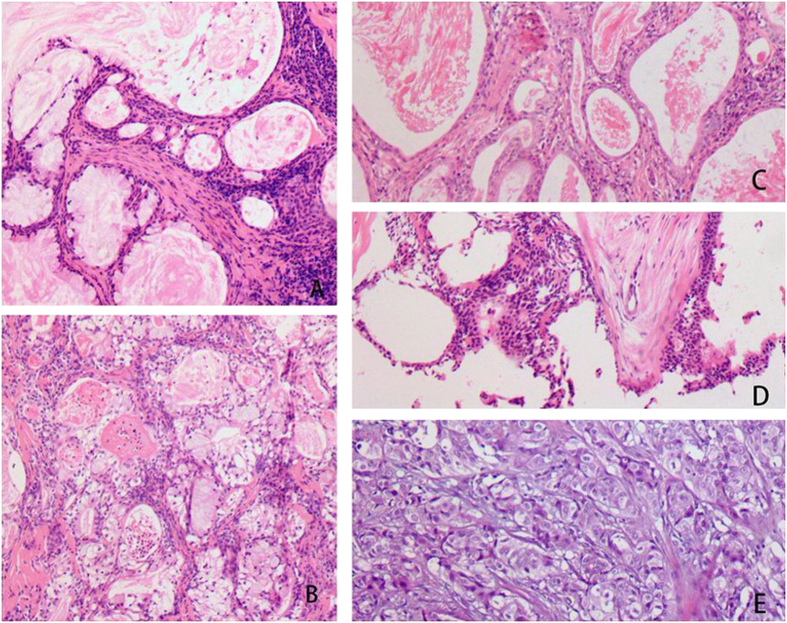
Histological characteristics of tumors in Case #1 (**A**) and #2 (**B**). Low-grade malignancies consisted of a large number of mucous cells and a small number of epidermoid cells. Tumors derived from Case #3 (**C**) and #4 (**D**): Low-grade malignancies were composed of a large number of epidermoid cells and a small number of mucous cells. Tumor derived from Case #5 (**E**) was high-grade malignancy contained a large number of epidermoid cells with anaplasia and a small number of mucous cells.

Warthin’s tumor was composed of large gland and microcystic lumen. The gland and the inside of the cystic walls were covered with two layers of epithelia, with columnar oncocytic cells in the inner layer and smaller basal cells in the outer layer. Oncocytic secretion could be observed in the glands and cystic lumens. MECs were identified within WTs in all five cases. MECs had abundant fibrous stroma with focal hyalinization and scarce lymphoid elements. MECs exhibited invasive growth into the lymphoid tissues of WTs, with fibrous stroma. In Cases #2 and 4, the invasion of nerve fibers by MECs was evident. In Cases #1, 4, and 5, the invasion of capsules by MECs was observed. In all five cases, transitional zones between WTs and MECs elements were distinguished (Fig. 3).

### IHC and H&E

**Figure 4:**
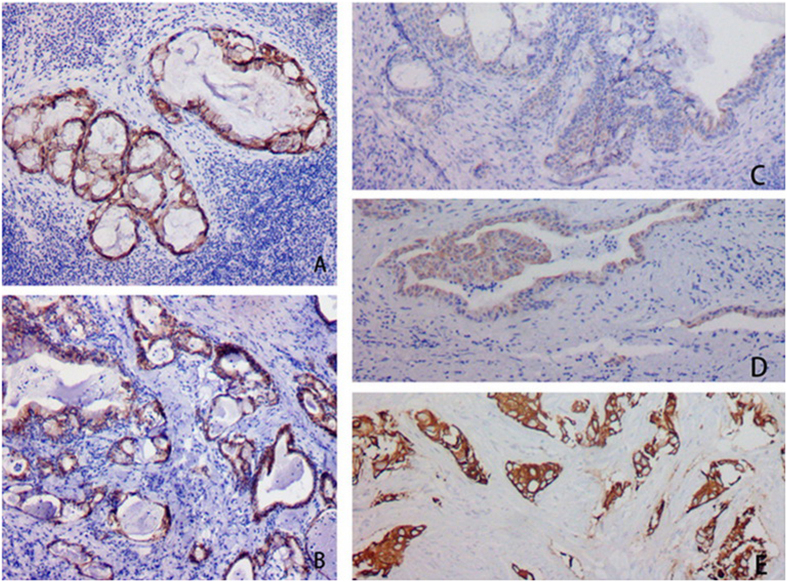
CK7 was positive in mucous and epidermoid cells in MECs.

**Figure 5:**
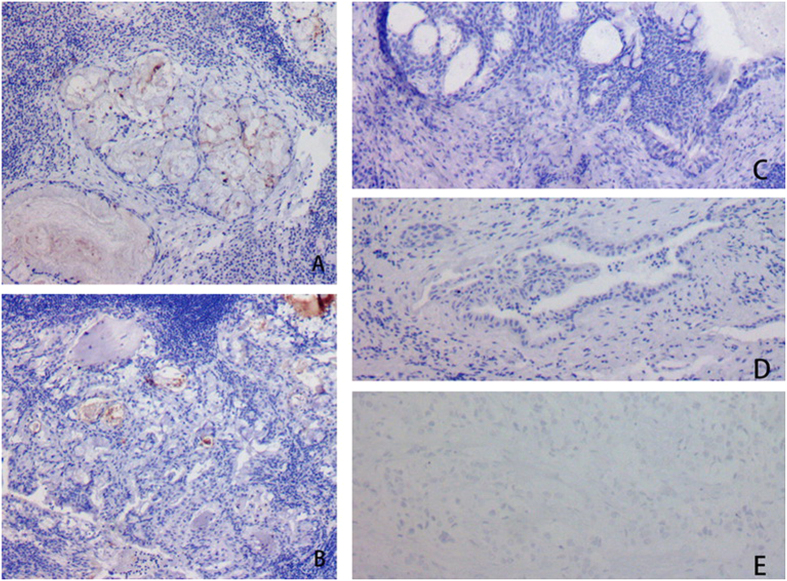
CEA was positive in mucous cells of MEC in Case #1 (**A**) and #2 (**B**) whereas negative in epidermoid cells of MEC. CEA was negative in mucous and epidermoid cells of MECs in Case #3 (**C**), #4 (**D**) and #5 (**E**).

**Figure 6:**
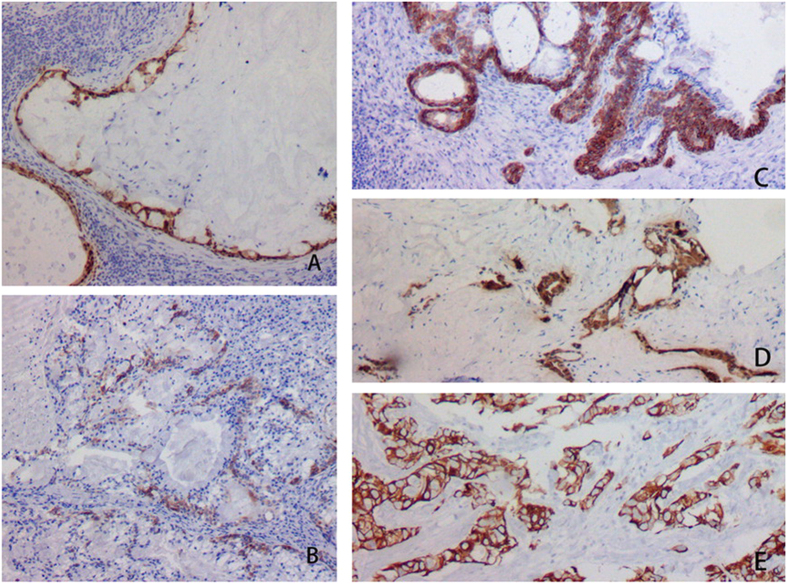
CK5/6 was highly overexpressed in epidermoid cells of MEC, whereas negative in mucous cells.

**Figure 7:**
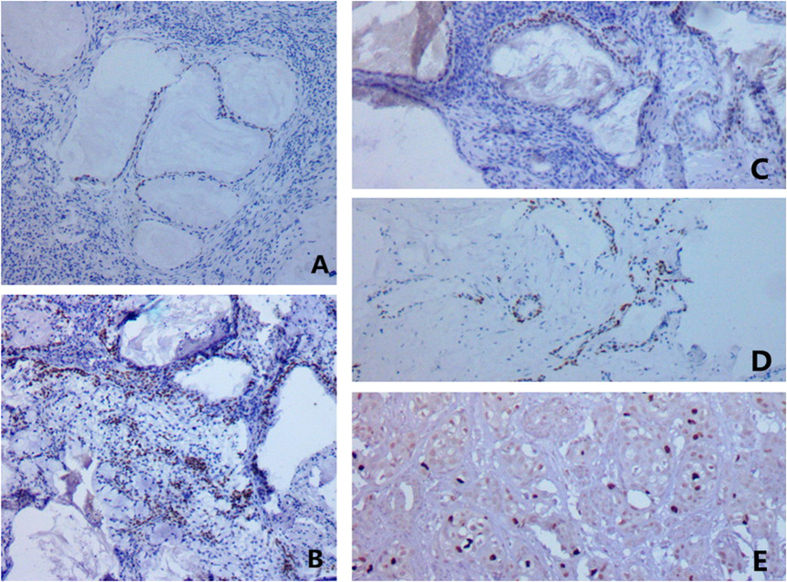
P63 was positive in epidermoid cells in MECs.

**Figure 8:**
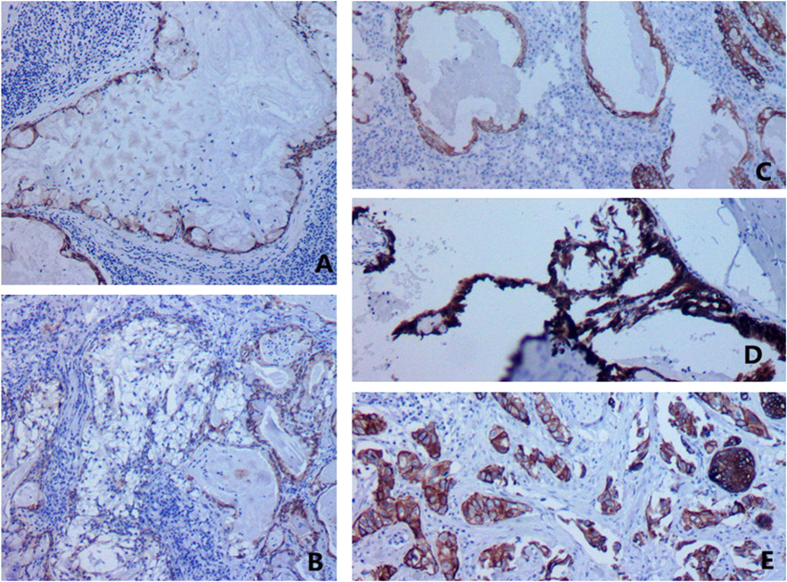
CK34βE12 was positive in epidermoid cells in MECs.

**Figure 9:**
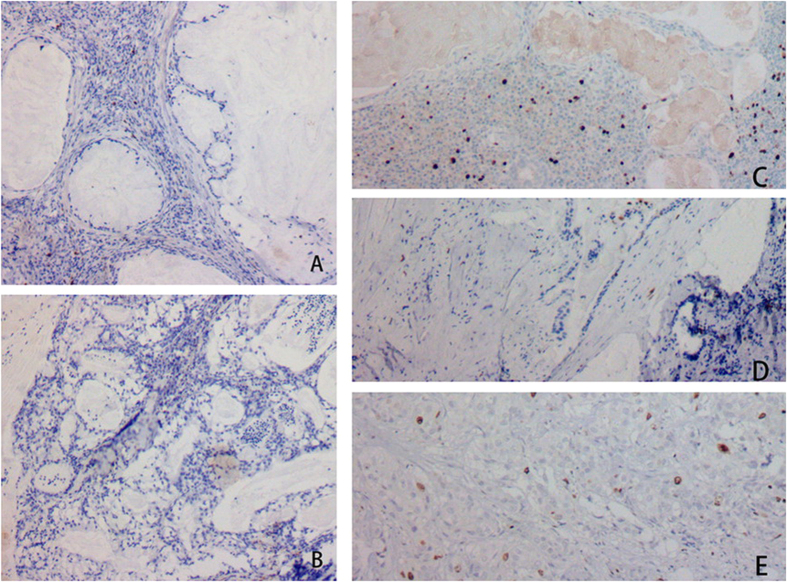
Ki67 index of MEC was 1% in Case #1 (**A**) #2 (**B**) and #3 (**C**); 2% in Case #4 (**D**); and 5% in Case #5 (**E**).

The oncocytic cells in the inner layer of WTs were positive CK5/6, CK34βE12, and CK7; whereas negative for P63, CEA, and CK20. The basal cells in the outer layer were positive for CK5/6, P63, and CK34βE12, whereas negative for CK7, CK20, and CEA. Mucous cells in MECs expressed CK7 (Fig. 4) and CEA (Fig. 5) whereas negative for CK5/6, P63, CK34βE12, and CK20. The epidermoid cells expressed CK5/6 (Fig. 6), P63 (Fig. 7), and CK34βE12 (Fig. 8) whereas negative for CK7, CK20, and CEA. For Case 4 and 5, Ki67 index in MEC was higher than that in WT (Fig. 9), (Table 2).

**Figure 10:**
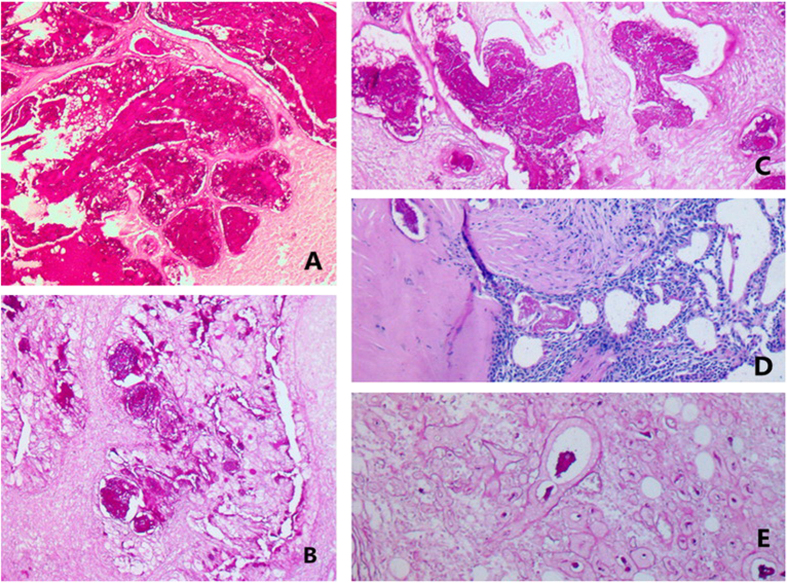
PAS was positive in mucous cells in MECs.

Periodic acid-Schiff (PAS) staining was positive in mucous cells of MECs. Mucus secretion from the cystic lumen was observed in WTs, while exudative mucus component was present in the stroma of MECs (Fig. 10).

### Follow up

MECs patients were followed up for 25–69 months. Currently, there is no local recurrence or distant metastasis.

## Discussion

The etiology of the malignant transformation of the epithelial component of Warthin’s tumor remains unclear. Local chronic inflammation, ischemia or hypoxic microenvironment may play important roles in mediating epithelial metaplasia to atypical hyperplasia^9^.

Warthin’s tumor occurs mostly in older males. In the reported cases of MEC arising from WT, the youngest one was a 35-year-old female^10^ and the oldest one was a 64-year-old male^11^. Age of onset of most patients was between 50–65 years, and the ratio between males and females was almost 1:1. There were 3 males and 2 females in the present study. The average age was 45.6 years, and the youngest was a 26-year-old female.

Warthin’s tumor occurs frequently in parotid glands and their lymph nodes, occasionally in minor salivary glands. WT is located in the tail lobe or deep lobe of parotid gland^9^. Classic clinical presentation is a slow-growing painless mass, although a few cases can cause pain or exhibit rapid growth^21^. If the mass increases suddenly, the possibility of malignancy or inflammation should be considered. Clinical manifestations of 5 patients in our study were similar to those reported in the literature. All patients presented painless masses in the parotid glands. Notably, in one case, the first symptom was facial paralysis. Facial paralysis is rare in WT, attributed to secondary inflammation, fibrosis, metaplasia^22^, or malignancy. MEC arising from WT was mostly a single nodular or oval mass located in lateral parotid gland^11^, rarely multiple nodules or located in bilateral parotid glands^5^. When MEC occurred in the interior part of WT, the capsule was clearly observed macroscopically. If MEC was located at the margin or invades the capsule, the boundary became ambiguous.

Cross sections of the masses were solid or cystic-solid, grayish-brown in color, and mucous. Cancerous region was usually desmoplastic, grayish-white in color. WT and MEC can co-exist as MEC is located in the interior of or adjacent to WT. Transitional zone between the epithelial component of WT and MEC confirms the diagnosis. It is difficult to distinguish metaplastic goblet cells of WT from mucous cells of a low-grade MEC^11^. In these 5 cases, oncocytic epithelium was present in mucous and squamous metaplasia of WT, while metaplastic goblet cells and epidermoid cells exhibited as a patchy distribution in MEC and mucous lake. Direct evidence of malignant transformation was infiltration of lymphoid tissues and encapsulation of WT by MEC^13^. WT and MEC had two types of epithelial components. WT consists of oncocytic and basal cells, whereas MEC consists of mucous and epidermoid cells. Basal cells of WT and epidermoid cells of MEC were both positive for CK5/6, CK34βE12 and P63. Oncocytic and mucous cells were both positive for CK7. Immunophenotypic features provided theoretical support for MEC arising from WT. Lymphoid tissues or the capsule infiltrated by MEC is typical. A transitional zone between the epithelial components of MEC and WT is essential for diagnosis.

MEC arising from WT should be differentiated from the following lesions. The first is WT accompanied by metaplasia of squamous cells and mucous cells. Similar to normal parotid gland, WT accompanied by focal squamous and mucous cells is not uncommon. Approximately 7.5% of WT has extensive squamous cell metaplasia^13^. Normal epithelial tissues in WT exhibit a double-layer structure; while abnormal structure and atypical cells appear with the metaplasia of squamous and mucous cells, characterized by an increase in epithelial layers, and the formation of cystic structures lined by mucous and intermediate cells. Therefore, it is difficult to determine the presence of malignant transformation. The key point of identification is that metaplasia lacks atypical cells and infiltrative growth.

Second, hyperplasia of stromal fibrous tissue and hyaline degeneration can be observed in MEC^12^. It is difficult to distinguish WT’s metaplastic mucous epithelium from MEC’s mucous epithelium based on dysplasia. In these 5 cases, mucous and oncocytic cells were mixed and distributed in mucous metaplasia of WT. Patchy distribution of mucous glands, irregular gland structure, mucous lakes, mucus spillover, and cancer stroma suggested the possibility of MEC. In future, more samples will be collected to identify evidence of malignant transformation.

Third, WT and MEC can develop independently and co-exist^9^. MEC arising from WT should be differentiated from another pattern of malignant transformation of WT, i.e., squamous cell carcinoma arising from WT. The key point of differential diagnosis is the presence of mucous cells, represented by positive CEA and AB/PAS staining. In addition, the epithelioid cells of MECs are rarely keratinized^8^.

Fourth, an extremely rare condition “tumor-to-tumor metastasis” should be considered. WT may act as a recipient of the malignancy that metastasizes from distant sites. It has been reported that lung carcinoma and renal carcinoma metastasize to WT^23^. The diagnosis is mainly based on the fact that other tumor is identified according to clinical manifestations and imaging results; and there is no transitional zone between the two tumors. The patients in this study had received comprehensive examinations. There was no tumor at other sites, while there was a transitional zone between the two tumors. The differential diagnosis between the malignant transformation of WT’s epithelial component and metastatic tumor is very important for the treatment and prognosis of the patients. If WT is combined with a metastatic tumor, local surgical resection alone is not adequate to achieve a satisfactory outcome.

The treatment of MEC arising in WT is mainly surgical operation, including resection of the primary tumor combined with neck lymph node dissection. MEC arising in WT is susceptible to recurrence or metastasis, which requires a long-term follow-up. In this study, 3 cases received surgery only, and 2 cases received surgery combined with radioactive particle implantation. So far, the reported cases of MEC arising from WT have been very limited, and most cases have no recurrence or distant metastases^11^,^13^. The 5 cases in the present study were followed up for 25–69 months. Currently, serum CEA, CA125, AFP and CA-199 levels are all in normal range. No recurrence or distant metastasis of the tumor has been found by imaging examination. A long-term prognosis still awaits further observations.

## Materials and Methods

### Patients

This study has been approved by Institutional Review Board (IRB) committees at Chinese PLA General Hospital and Beijing Shijitan Hospital. Informed consent was acquired from each participate before the operation. All procedures were conducted according to the guidelines approved by the ethics committees at Chinese PLA General Hospital and Beijing Shijitan Hospital. Among 309 cases of consecutively, surgically resected Warthin tumors at The Chinese People’s Liberation Army (PLA) General Hospital (n = 286, recruited from 2009 to 2014) and Beijing Shijitan Hospital of Capital Medical University (n = 23, recruited from 2010 to 2014), 5 cases fulfilled the criteria for MECs arising from WTs. None of the cases had MECs at any other sites at the time of diagnosis or during the follow-up after surgery.

### Immunohistochemistry (IHC)

The samples were conventionally fixed in 10% neutral buffered formalin. For light microscopy, sections were stained with hematoxylin and eosin (H&E), periodic acid-Schiff (PAS) without diastase digestion and Alcian blue. For IHC assays, the following markers were used: cytokeratin 5/6 (CK5/6), CK34βE12 (high molecular weight cytokeratins 1, 5, 10 and 14), CK7, CK20, carcinoembryonic antigen (CEA), P53, P63, and Ki-67. All antibodies and reagents were purchased from DakoCytomation (Glostrup, Denmark). Briefly, the tissue sections were deparaffinized in xylene and rehydrated through a series of graded alcohols. Endogenous peroxidase activity was blocked in a solution containing 0.3% hydrogen peroxide. Then the sections were boiled in diluted EnVision FLEX Target Retrieval solution, for 20 min in a Water Bath. After cooling down to room temperature, the sections were incubated with the primary antibody for 30 min at room temperature. Immunoreactivity was revealed by application of the Rabbit/Mouse EnVision reagent (30 min) and DAB (5 min). Finally, the slides were counterstained with hematoxylin solution and mounted.

## Additional Information

**How to cite this article**: Yu, C. *et al*. Mucoepidermoid carcinoma arising in Warthin’s tumor of the parotid gland: Clinicopathological characteristics and immunophenotypes. *Sci. Rep.* **6**, 30149; doi: 10.1038/srep30149 (2016).

## Figures and Tables

**Table 1:** Characteristics of MECs arising from Warthin’s tumor of the parotid gland.

**ID**	**Gender**	**Age**	**Location**	**Presentation**	**Tumor size (cm)**	**Treatment**	**Follow-up**
1	Female	43	Superficial lobe of RPD	PPM for 9 months	3.6	Resection, radioactive seeds implantation	25 months, NORM
2	Male	40	Superficial lobe of RPD	PPM for 3 months	4.2	Resection	48 months, NORM
3	Male	63	Superficial lobe of LPD	PPM for 50 days	2.0	Resection	63 months, NORM
4	Female	26	Superficial lobe of RPD	PPM for 3 years	2.5	Resection, radioactive seeds implantation	69 months, NORM
5	Male	56	Deep lobe of RPD	PPM, facial paralysis for 9 months	Multiple (0.5–2.5)	Resection	25 months, NORM

RPD: right parotid gland

PPM: presented painless masses

NORM: no evidence of recurrence or metastasis.

**Table 2:** Biomarkers of MECs arising from Warthin’s tumor of the parotid gland.

**ID**	**CK7**	**CK20**	**CEA**	**CK5/6**	**P63**	**CK34β12**	**P53**	**Ki-67 Index**
1	oncocytic cells of WT, Mucous and epidermoid cells in MEC (+)	(−)	mucous cells in MEC (+)	oncocytic and basal cells of WT, epidermoid cells of MEC (+), mucous cells (−)	basal cells of WT, epidermoid cells of MEC (+)	oncocytic and basal cells of WT, epidermoid cells of MEC (+), mucous cells (−)	(−)	1% in WT and MEC
2	oncocytic cells of WT, Mucous and epidermoid cells in MEC (+)	(−)	mucous cells in MEC (+)	oncocytic and basal cells of WT, epidermoid cells of MEC (+), mucous cells (−)	basal cells of WT, epidermoid cells of MEC (+)	oncocytic and basal cells of WT, epidermoid cells of MEC (+), mucous cells (−)	(−)	1% in WT and MEC
3	oncocytic cells of WT, Mucous and epidermoid cells in MEC, focal (+)	(−)	(−)	oncocytic and basal cells of WT, epidermoid cells of MEC (+), mucous cells (−)	basal cells of WT, epidermoid cells of MEC (+)	oncocytic and basal cells of WT, epidermoid cells of MEC (+), mucous cells (−)	(−)	1% in WT and MEC
4	oncocytic cells of WT, Mucous and epidermoid cells in MEC (+)	(−)	(−)	oncocytic and basal cells of WT, epidermoid cells of MEC (+), mucous cells (−)	basal cells of WT, epidermoid cells of MEC (+)	oncocytic and basal cells of WT, epidermoid cells of MEC (+), mucous cells (−)	(−)	1% in WT, 2% in MEC
5	oncocytic cells of WT, Mucous and epidermoid cells in MEC (+)	(−)	(−)	oncocytic and basal cells of WT, epidermoid cells of MEC (+), mucous cells (−)	basal cells of WT, epidermoid cells of MEC (+)	oncocytic and basal cells of WT, epidermoid cells of MEC (+), mucous cells (−)	(−)	1% in WT, 5% in MEC
